# Yaws Disease Caused by *Treponema pallidum* subspecies *pertenue* in Wild Chimpanzee, Guinea, 2019

**DOI:** 10.3201/eid2606.191713

**Published:** 2020-06

**Authors:** Benjamin Mubemba, Emeline Chanove, Kerstin Mätz-Rensing, Jan F. Gogarten, Ariane Düx, Kevin Merkel, Caroline Röthemeier, Andreas Sachse, Helene Rase, Tatyana Humle, Guillaume Banville, Marine Tchoubar, Sébastien Calvignac-Spencer, Christelle Colin, Fabian H. Leendertz

**Affiliations:** Copperbelt University, Kitwe, Zambia (B. Mubemba);; Robert Koch Institute, Berlin, Germany (B. Mubemba, J.F. Gogarten, A. Düx, K. Merkel, C. Röthemeier, A. Sachse, S. Calvignac-Spencer, F.H. Leendertz);; University of Agricultural Sciences and Veterinary Medicine, Cluj-Napoca, Romania (E. Chanove);; Chimpanzee Conservation Center, Somoria, Faranah, Republic of Guinea (E. Chanove, H. Rase, G. Banville, M. Tchoubar, C. Colin);; Leibniz Institute for Primate Research, Göttingen, Germany (K. Mätz-Rensing);; University of Kent, Canterbury, UK (T. Humle)

**Keywords:** yaws disease, chimpanzee, Treponema pallidum, bacteria, Guinea, Treponema pallidum subspecies pertenue

## Abstract

Yaws-like lesions are widely reported in wild African great apes, yet the causative agent has not been confirmed in affected animals. We describe yaws-like lesions in a wild chimpanzee in Guinea for which we demonstrate infection with *Treponema pallidum* subsp. *pertenue*. Assessing the conservation implications of this pathogen requires further research.

Several monkey species in sub-Saharan Africa are infected with *Treponema pallidum* subspecies *pertenue* (TPE) and typically manifest yaws-like lesions on the face and distal extremities or syphilis-like lesions in the anogenital region (*1*). Reports of nonhuman primates (NHPs) infected with TPE came from West Africa in the 1960s. These studies were based on seroprevalence studies finding that yellow baboons (*Papio cynocephalus cynocephalus*) had a 60% seroprevalence rate for treponemal-specific antibodies (*2*,*3*). Whole-genome sequencing of the isolate collected from these baboons later revealed similarities with TPE causing yaws in humans (*3*,*4*). In the late 1980s in Gombe National Park in Tanzania, olive baboons (*Papio anubis*) with genital ulcerations were found to have yaws-like infections of the skin (*5*). Later genetic and serologic studies confirmed infections with *T. pallidum* in olive baboons at many sites in Tanzania (*6**,**7*). 

Both genital and orofacial lesions attributable to TPE infection have been documented in several NHP species across sub-Saharan Africa (African green monkeys [*Chlorocebus sabaeus*] in Bijilo Forest Park, The Gambia, and Niokola-Koba National Park, Senegal; sooty mangabeys [*Cercocebus atys atys*] in Taï National Park, Côte d’Ivoire) (*1*; B. Mubemba et al., unpub. data, ). Two studies observed that TPE infections remain geographically widespread in Tanzania and affect olive baboons, yellow baboons, vervet monkeys (*Chlorocebus pygerythrus*), and blue monkeys (*Cercopithecus mitis*), as well as grivet monkeys (*Chlorocebus aethiops*) from Ethiopia (*8*,*9*).

Symptoms and skeletal deformation have also been observed in great apes, specifically gorillas (*Gorilla gorilla*) in the Republic of Congo, Gabon, and Cameroon (*10*), as well as in chimpanzees (*Pan troglodytes*) in Cameroon, Uganda, and Côte d’Ivoire, and are suggestive of TPE infections (*10*; F.H. Leendertz, pers. comm., 2019 Nov 1), but matching diagnostics are currently unavailable. The only diagnostic evidence is based on TPE DNA from 2 chimpanzee (*P. troglodytes verus*) bones (*11*) and gorilla feces (*9*) of unknown individual great apes, so no link between diagnostics and clinical signs can be made. We present matching clinical and molecular evidence of TPE infection in a wild great ape.

## The Study

**Figure 1:**
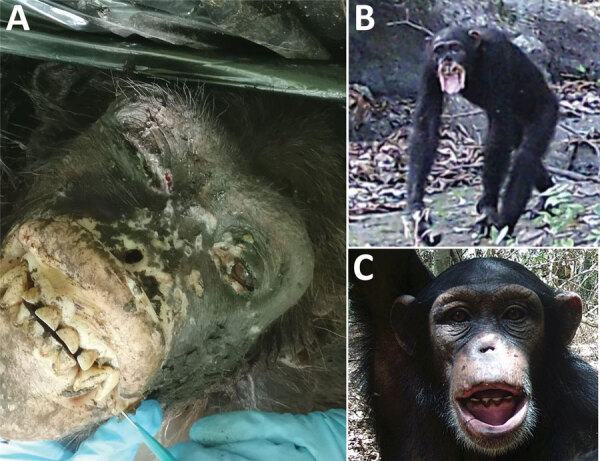
Yaws-like lesions in wild chimpanzees, Guinea. A) Yaws-like lesions observed during a necropsy of an adult female chimpanzee found in the Sangaredi area, Guinea. B, C) Camera trap images showing yaws-like lesions on adult (B) and juvenile (C) chimpanzees in Haut Niger National Park, Guinea.

We found a cachectic wild adult female chimpanzee (*Pan troglodytes verus*) with severe yaws-like lesions on the mouth and lips in a mining concession in Sangaredi area, Guinea (Figure 1, panel A). The chimpanzee was in visible agony and had to be euthanized; we performed a necropsy on the body. Gross pathology of the skin revealed a marked depigmentation on hypertrophied edematous lips; crusts and ulcers were present on the head, and much of the nose was missing. The eyes were shrunken and purulent and surrounded by crusts, and the corneas were opaque. We preserved samples of lesioned skin in 10% formalin and RNAlater (Thermo Fisher, https://www.thermofisher.com).

**Figure 2:**
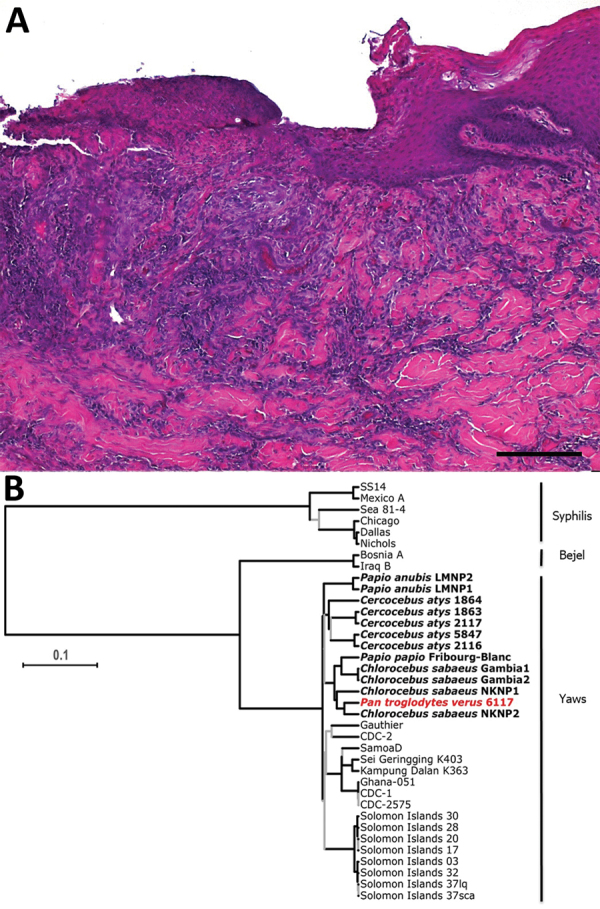
Histopathologic analysis of yaws-like lesions in a wild chimpanzee, Guinea, and phylogenetic placement of the *Treponema pallidum* subspecies *pertenue* strain. A) Histopathologic evidence suggestive of a treponemal infection. Shown here is superficial ulcerative pyogranulomatous dermatitis including formation of a mixed inflammatory cell infiltration, predominantly neutrophil granulocytes. Deeper dermal layers show the formation of a perivascular lymphocytic inflammatory cell infiltrate, focal folliculitis, and perifolliculitis. Skin areas adjacent to ulcerated parts show irregular epidermal hyperplasia, consistent with treponemal infections. The ulcerated areas were covered by a serocellular crust. Scale bar indicates 200 μm. B) Maximum clade credibility tree of *T. pallidum* strain genomes. Red indicates the chimpanzee genome generated in this study. All simian-infecting strains are shown in bold with labels showing the species of nonhuman primate, and the diseases caused by each type of bacteria are shown at right. Branches supported by posterior probabilities <0.95 in the Bayesian Markov chain Monte Carlo tree are indicated in gray. Scale bar indicates nucleotide substitutions per variable site.

We analyzed formalin-fixed skin samples with both histological and immunohistochemical methods, as previously described (*6*). Histopathological features of the skin biopsies were compatible with treponemal infection (Figure 2, panel A). Skin lesions were characterized by irregular epidermal proliferation of different extents. The epidermis developed hyperkeratosis and hypertrophy of the epidermal rete pegs, which branched and projected deeply into the corium. Admixed areas with severe superficial erosions or deep ulcerations were visible. A moderate to severe mixed cell infiltration composed of lymphocytes and histiocytes was present in the underlying dermal layer. The cellular reaction was most pronounced around the dermal blood vessels and hair follicles, resulting in superficial and deep perivascular dermatitis. The epidermal surface was covered with a dried serosanguineous discharge. Immunohistochemical analyses failed to visualize treponemes, which is a frequent problem resulting from low numbers of bacteria at lesion sites (*6*).

We extracted DNA from 2 facial lesion biopsies stored in RNAlater and performed molecular investigations (Appendix). High-throughput sequencing analysis resulted in a 24-fold average coverage of the TPE genome; 98.6% of the genome was covered by >1 read and 97.6% by >3 reads. Bayesian Markov chain Monte Carlo analysis of a genomic alignment comprising this reconstructed TPE genome, all other available TPE and *Treponema pallidum* subsp. *endemicum* (TEN, bejel) genomes, and a selection of *Treponema pallidum* subsp. *pallidum* (TPA, syphilis) genomes available in Genbank (Appendix Table) revealed that the chimpanzee-derived genome clustered within the well-supported TPE clade, indicating that TPE is responsible for the clinical picture observed in this particular wild chimpanzee (Figure 2, panel B). More precisely, this new chimpanzee-derived genome belongs to a clade consisting of TPE strains isolated from NHPs in far western Africa in Gambia, Guinea Bissau, Senegal, and Guinea, in agreement with recent observations that genomic diversity of TPE strains infecting NHPs appears to be geographically structured (*9*; B. Mubemba et al., unpub. data, ). Yaws is principally a skin disease, and it seems likely that the poor condition of this animal was caused by another unknown but likely traumatic cause, perhaps coupled with associated secondary infections, although our field necropsy was not able to identify an alternative cause of her cachectic condition.

To determine whether TPE might affect other chimpanzees in Guinea, we examined videos collected by camera traps set near the Chimpanzee Conservation Center in Haut Niger National Park. During 2018–2019, in 10 different camera trap locations, we observed 12 chimpanzees (1 juvenile, 3 subadults, and 8 adults) with severe lesions. The lesions observed in these images closely resembled those of the wild female from the Sangaredi region described in this article, including shrunken eyes, deformation of the face, absence of the nose, and hypertrophied and depigmented lips (in 1 case, the lips were completely missing; Figure 1, panels B, C). Molecular investigations of the pathogen(s) causing these infections is clearly warranted, perhaps through noninvasive screening of TPE in feces, bones, or primate-associated flies (*9*,*12*).

## Conclusions

This study links yaws-like pathology to the actual detection of TPE in a wild chimpanzee, providing evidence that at least part of the suggestive lesions often observed in wild great apes are caused by this pathogen. These data join a growing body of evidence demonstrating that many NHP species across sub-Saharan Africa are infected with TPE (*1*,*9*). This finding could potentially be problematic for the ongoing campaign to eradicate TPE globally by 2030 (*13*), although, clearly, data from TPE-infected humans in this region are needed to determine whether zoonotic transmission of this pathogen occurs. Given the severity of lesions, it is evident that individual animal fitness is affected. The impact of this disease on NHP populations is unknown but could be assessed through long-term monitoring.

Appendix Additional information on molecular and bioinformatics analysis of *Treponema pallidum* in a wild chimpanzee, Guinea.
