# Supplementary material for: Yaws Disease Caused by Treponema pallidum subspecies pertenue in Wild Chimpanzee, Guinea, 2019
Source: Emerg Infect Dis. 2020 Jun;26(6):1283–6. doi: 10.3201/eid2606.191713 (PMC7258472; doi:10.3201/eid2606.191713)
Supplement: Appendix — Additional information on molecular and bioinformatics analysis of Treponema pallidum in a wild chimpanzee, Guinea. [file 19-1713-Techapp-s1.pdf]

DOI: https://doi.org/10.3201/eid2606.191713

Yaws Disease Caused by *Treponema pallidum* subspecies *pertenue* in Wild Chimpanzee, Guinea, 2019

Appendix

Molecular and Bioinformatics Analyses

We extracted DNA from 2 chimpanzee facial lesion biopsies using the DNeasy blood and tissue extraction kit (QIAGEN, https://www.qiagen.com) following the manufacturer's protocol. We screened samples with a quantitative PCR targeting the *pol*A gene of *Treponema pallidum*, as described previously (*1*); both samples were positive. We converted DNA extracts into dual indexed Illumina libraries using the NEBNext Ultra II DNA Library Prep kit (New England Biolabs, https://www.neb.com). We enriched the libraries for TPE through in-solution hybridization capture, as previously described (*2*; B. Mubemba et al., unpub. data, https://doi.org/10.1101/848382) sequenced on an Illumina NextSeq (v2 chemistry, 2x150 cycles; https://www.illumina.com). We quality-filtered the reads using Trimmomatic v0.38 (removing leading and trailing reads <Q30; clipping reads where average base quality across 4 bp was <30; removing surviving reads <30 nt long) (*3*). We merged surviving read pairs with Clip and Merge v1.7.8 (https://anaconda.org/bioconda/clipandmerge). We combined merged reads and surviving singletons and mapped them to TPE Fribourg-Blanc (RefSeq ID NC_021179.1) using BWA-MEM with a minimum seed length of 29. We sorted mapped reads using Picard's SortSam, de-duplicated them with Picard's MarkDuplicates (https://broadinstitute.github.io/picard/index.html), and removed alignments with MAPQ <30 and a mapping length <30 nt using SAMtools (*4*). Finally, we merged all mapped reads of individual library samples to produce single TPE draft genome. We used Geneious v.11 to call a consensus genome requiring a minimum of 3 unique reads to cover a position for it to be called and applying a majority consensus rule (*5*).

We performed whole genome alignment using the multiple sequence alignment program MAFFT (*6*). We then removed all putative recombinant genes (*2*) and selected conserved blocks using the Gblocks tool (*7*) in SeaView v4 (*8*). We performed Bayesian Markov chain Monte Carlo phylogenomic analysis in BEAST (version 1.10.4; https://www.mybiosoftware.com) on the resulting alignment of 4,213 variable positions (after stripping off of all ambiguities and identical sites in the final dataset); settings of the analysis were a strict clock model and a coalescent process assuming constant population size. We examined the output of multiple chains of 10,000,000 generations for convergence and appropriate sampling of the posterior using Tracer (version 1.7.1) (*9*) before merging tree files using Log Combiner (version 1.10.4) (*10*). We picked the best representative tree from the posterior set of trees and annotated it with Tree Annotator (version 1.10.4: distributed with BEAST). We further edited the resultant maximum clade credibility (MCC) tree file using iTOL (https://itol.embl.de) (*11*). *T. pallidum* sequences used in this study are listed in the Appendix Table (*12–24*).

References

1. Leslie DE, Azzato F, Karapanagiotidis T, Leydon J, Fyfe J. Development of a real-time PCR assay to detect *Treponema pallidum* in clinical specimens and assessment of the assay's performance by comparison with serological testing. J Clin Microbiol. 2007;45:93–6. PubMed https://doi.org/10.1128/JCM.01578-06

2. Arora N, Schuenemann VJ, Jäger G, Peltzer A, Seitz A, Herbig A, et al. Origin of modern syphilis and emergence of a pandemic *Treponema pallidum* cluster. Nat Microbiol. 2017;2:16245. PubMed https://doi.org/10.1038/nmicrobiol.2016.245

3. Bolger AM, Lohse M, Usadel B. Trimmomatic: a flexible trimmer for Illumina sequence data. Bioinformatics. 2014;30:2114–20. PubMed https://doi.org/10.1093/bioinformatics/btu170

4. Li H, Handsaker B, Wysoker A, Fennell T, Ruan J, Homer N, et al.; 1000 Genome Project Data Processing Subgroup. The sequence alignment/map format and SAMtools. Bioinformatics. 2009;25:2078–9. PubMed https://doi.org/10.1093/bioinformatics/btp352

5. Kearse M, Moir R, Wilson A, Stones-Havas S, Cheung M, Sturrock S, et al. Geneious Basic: an integrated and extendable desktop software platform for the organization and analysis of sequence data. Bioinformatics. 2012;28:1647–9. 10.1093/bioinformatics/bts199 PubMed https://doi.org/10.1093/bioinformatics/bts199

6. Katoh K, Standley DM. MAFFT multiple sequence alignment software version 7: improvements in performance and usability. Mol Biol Evol. 2013;30:772–80. PubMed https://doi.org/10.1093/molbev/mst010

7. Talavera G, Castresana J. Improvement of phylogenies after removing divergent and ambiguously aligned blocks from protein sequence alignments. Syst Biol. 2007;56:564–77. https://doi.org/10.1080/10635150701472164

8. Gouy M, Guindon S, Gascuel O. SeaView version 4: A multiplatform graphical user interface for sequence alignment and phylogenetic tree building. Mol Biol Evol. 2010;27:221–4. PubMed https://doi.org/10.1093/molbev/msp259

9. Rambaut A, Drummond AJ, Xie D, Baele G, Suchard MA. Posterior summarization in Bayesian phylogenetics using Tracer 1.7. Syst Biol. 2018;67:901–4. PubMed https://doi.org/10.1093/sysbio/syy032

10. Drummond AJ, Rambaut A. BEAST: Bayesian evolutionary analysis by sampling trees. BMC Evol Biol. 2007;7:214. PubMed https://doi.org/10.1186/1471-2148-7-214

11. Letunic I, Bork P. Interactive Tree Of Life (iTOL) v4: recent updates and new developments. Nucleic Acids Res. 2019;47(W1):W256–9. PubMed https://doi.org/10.1093/nar/gkz239

12. Štaudová B, Strouhal M, Zobaníková M, Čejková D, Fulton LL, Chen L, et al. Whole genome sequence of the *Treponema pallidum* subsp. *endemicum* strain Bosnia A: the genome is related to yaws treponemes but contains few loci similar to syphilis treponemes. PLoS Negl Trop Dis. 2014;8:e3261. PubMed https://doi.org/10.1371/journal.pntd.0003261

13. Mikalová L, Strouhal M, Oppelt J, Grange PA, Janier M, Benhaddou N, et al. Human *Treponema pallidum* 11q/j isolate belongs to subsp. *endemicum* but contains two loci with a sequence in TP0548 and TP0488 similar to subsp. *pertenue* and subsp. *pallidum*, respectively. PLoS Negl Trop Dis. 2017;11:e0005434. PubMed https://doi.org/10.1371/journal.pntd.0005434

14. Pětrošová H, Pospíšilová P, Strouhal M, Čejková D, Zobaníková M, Mikalová L, et al. Resequencing of *Treponema pallidum* ssp. *pallidum* strains Nichols and SS14: correction of sequencing errors resulted in increased separation of syphilis treponeme subclusters. PLoS One. 2013;8:e74319. PubMed https://doi.org/10.1371/journal.pone.0074319

15. Giacani L, Jeffrey BM, Molini BJ, Le HT, Lukehart SA, Centurion-Lara A, et al. Complete genome sequence and annotation of the *Treponema pallidum* subsp. *pallidum* Chicago strain. J Bacteriol. 2010;192:2645–6. PubMed https://doi.org/10.1128/JB.00159-10

16. Zobaníková M, Mikolka P, Cejková D, Pospíšilová P, Chen L, Strouhal M, et al. Complete genome sequence of *Treponema pallidum* strain DAL-1. Stand Genomic Sci. 2012;7:12–21. PubMed https://doi.org/10.4056/sigs.2615838

17. Giacani L, Iverson-Cabral SL, King JCK, Molini BJ, Lukehart SA, Centurion-Lara A. Complete genome sequence of the *Treponema pallidum* subsp. *pallidum* Sea81–4 strain. Genome Announc. 2014;2:e00333-14. PubMed https://doi.org/10.1128/genomeA.00333-14

18. Zobaníková M, Strouhal M, Mikalová L, Čejková D, Ambrožová L, Pospíšilová P, et al. Whole genome sequence of the *Treponema* Fribourg-Blanc: unspecified simian isolate is highly similar to the yaws subspecies. PLoS Negl Trop Dis. 2013;7:e2172. PubMed https://doi.org/10.1371/journal.pntd.0002172

19. Čejková D, Zobaníková M, Chen L, Pospíšilová P, Strouhal M, Qin X, et al. Whole genome sequences of three *Treponema pallidum* ssp. *pertenue* strains: yaws and syphilis treponemes differ in less than 0.2% of the genome sequence. PLoS Negl Trop Dis. 2012;6:e1471. PubMed https://doi.org/10.1371/journal.pntd.0001471

20. Liska SL, Perine PL, Hunter EF, Crawford JA, Feeley JC. Isolation and transportation of *Treponema pertenue* in golden hamsters. Curr Microbiol. 1982;7:41–3. https://doi.org/10.1007/BF01570978

21. Strouhal M, Mikalová L, Havlíčková P, Tenti P, Čejková D, Rychlík I, et al. Complete genome sequences of two strains of *Treponema pallidum* subsp. *pertenue* from Ghana, Africa: identical genome sequences in samples isolated more than 7 years apart. PLoS Negl Trop Dis. 2017;11:e0005894. PubMed https://doi.org/10.1371/journal.pntd.0005894

22. Strouhal M, Mikalová L, Haviernik J, Knauf S, Bruisten S, Noordhoek GT, et al. Complete genome sequences of two strains of *Treponema pallidum* subsp. *pertenue* from Indonesia: Modular structure of several treponemal genes. PLoS Negl Trop Dis. 2018;12:e0006867. PubMed https://doi.org/10.1371/journal.pntd.0006867

23. Marks M, Fookes M, Wagner J, Butcher R, Ghinai R, Sokana O, et al. Diagnostics for yaws eradication: insights from direct next-generation sequencing of cutaneous strains of *Treponema pallidum.* Clin Infect Dis. 2018;66:818–24. PubMed https://doi.org/10.1093/cid/cix892

24. Knauf S, Gogarten JF, Schuenemann VJ, De Nys HM, Düx A, Strouhal M, et al. Nonhuman primates across sub-Saharan Africa are infected with the yaws bacterium *Treponema pallidum* subsp. *pertenue*. Emerg Microbes Infect. 2018;7:1–4. PubMed https://doi.org/10.1038/s41426-018-0156-4

Appendix Table. Published *Treponema pallidum* (TP) sequences used in the study of *T. pallidum* in wild chimpanzees.

Isolate ID	GenBank accession no.	Host	TP spectrum	Country	Reference
Bosnia A	CP007548.1	*Homo sapiens*	Bejel	Bosnia	(*12*)
Iraq_B	CP032303.1	*Homo sapiens*	Bejel	Iraq	(*13*)
Nichols	NC_021490.2	*Homo sapiens*	Syphilis	USA	(*14*)
SS14	NC_021508.1	*Homo sapiens*	Syphilis	USA	(*14*)
Chicago	NC_017268.1	*Homo sapiens*	Syphilis	USA	(*15*)
Mexico A	NC_018722.1	*Homo sapiens*	Syphilis	Mexico	(*14*)
Dallas	NC_016844.1	*Homo sapiens*	Syphilis	USA	(*16*)
Seattle 81–4	CP003679.1	*Homo sapiens*	Syphilis	USA	(*17*)
Fribourg-Blanc	NC_021179.1	*Papio papio*	Yaws	Guinea	(*18*)
Samoa D	NC_016842.1	*Homo sapiens*	Yaws	Samoa	(*19*)
Gauthier	NC_016843.1	*Homo sapiens*	Yaws	Republic of the Congo	(*19*)
CDC-1	CP024750.1	*Homo sapiens*	Yaws	Ghana	(*20*)
CDC-2	NC_016848.1	*Homo sapiens*	Yaws	Ghana	(*19*)
CDC_2575	CP020366	*Homo sapiens*	Yaws	Ghana	(*21*)
Ghana-051	CP020365	*Homo sapiens*	Yaws	Ghana	(*21*)
Kampung_Dalan_K363	CP024088.1	*Homo sapiens*	Yaws	Indonesia	(*22*)
Sei_Geringging_K403	CP024089.1	*Homo sapiens*	Yaws	Indonesia	(*22*)
Solomon Islands 03	ERR1470343	*Homo sapiens*	Yaws	Solomon Islands	(*23*)
Solomon Islands 17	ERR1470344	*Homo sapiens*	Yaws	Solomon Islands	(*23*)
Solomon Islands 20	ERR1470335	*Homo sapiens*	Yaws	Solomon Islands	(*23*)
Solomon Islands 28	ERR1470338	*Homo sapiens*	Yaws	Solomon Islands	(*23*)
Solomon Islands 30	ERR1470334	*Homo sapiens*	Yaws	Solomon Islands	(*23*)
Solomon Islands 32	ERR1470342	*Homo sapiens*	Yaws	Solomon Islands	(*23*)
Solomon Islands 37 liq	ERR1470330	*Homo sapiens*	Yaws	Solomon Islands	(*23*)
Solomon Islands 37 sca	ERR1470331	*Homo sapiens*	Yaws	Solomon Islands	(*23*)
Gambia-1	SRR4308597	*Chlorocebus sabeus*	Yaws	Gambia	(*24*)
Gambia-2	SRR4308605	*Chlorocebus sabaeus*	Yaws	Gambia	(*24*)
Senegal NKNP-1	SRR4308606	*Chlorocebus sabaeus*	Yaws	Senegal	(*24*)
Senegal NKNP-2	SRR4308607	*Chlorocebus sabaeus*	Yaws	Senegal	(*24*)
LMNP-1	CP021113.1	*Papio anubis*	Yaws	Tanzania	(*24*)
LMNP-2_BS5	SRR4308598	*Papio anubis*	Yaws	Tanzania	(*24*)
LMNP-2_BS6	SRR4308599	*Papio anubis*	Yaws	Tanzania	(*24*)
LMNP-2_BS7	SRR4308601	*Papio anubis*	Yaws	Tanzania	(*24*)
LMNP-2_BS8	SRR4308602	*Papio anubis*	Yaws	Tanzania	(*24*)
1863-Hato	SRR4308604	*Cercocebus atys*	Yaws	Côte d'Ivoire	(*24*)
1864-IGU	SRR4308596	*Cercocebus atys*	Yaws	Côte d'Ivoire	(*24*)
2117-BAK	SAMN13258074	*Cercocebus atys*	Yaws	Côte d'Ivoire	Mubemba et al.*
2116-OKA	SAMN13258075	*Cercocebus atys*	Yaws	Côte d'Ivoire	Mubemba et al.*
5847-CHAT	SAMN13258076	*Cercocebus atys*	Yaws	Côte d'Ivoire	Mubemba et al.*

*B. Mubemba et al., unpub. data, https://doi.org/10.1101/848382.
